# Evidence for association of *Vibrio echinoideorum* with tissue necrosis on test of the green sea urchin *Strongylocentrotus droebachiensis*

**DOI:** 10.1038/s41598-022-08772-2

**Published:** 2022-03-22

**Authors:** Jonathan Hira, Klara Stensvåg

**Affiliations:** The Norwegian College of Fishery Science, The Faculty of Biosciences, Fisheries and Economics, UiT The Arctic University of Norway, Tromsø, Norway

**Keywords:** Microbiology, Pathogens

## Abstract

“Sea urchin lesion syndrome” is known as sea urchin disease with the progressive development of necrotic epidermal tissue and loss of external organs, including appendages on the outer body surface. Recently, a novel strain, *Vibrio echinoideorum* has been isolated from the lesion of green sea urchin (*Strongylocentrotus droebachiensis*), an economically important mariculture species in Norway. *V. echinoideorum* has not been reported elsewhere in association with green sea urchin lesion syndrome. Therefore, in this study, an immersion based bacterial challenge experiment was performed to expose sea urchins (wounded and non-wounded) to *V. echinoideorum*, thereby mimicking a nearly natural host–pathogen interaction under controlled conditions. This infection experiment demonstrated that only the injured sea urchins developed the lesion to a significant degree when exposed to *V. echinoideorum*. Pure cultures of the employed bacterial strain were recovered from the infected animals and its identity was confirmed by the MALDI-TOF MS spectra profiling. Additionally, the hemolytic phenotype of *V. echinoideorum* substantiated its virulence potential towards the host, and this was also supported by the cytolytic effect on red spherule cells of sea urchin. Furthermore, the genome sequence of *V. echinoideorum* was assumed to encode potential virulence genes and were subjected to in silico comparison with the established virulence factors of *Vibrio vulnificus* and *Vibrio tasmaniensis*. This comparative virulence profile provided novel insights about virulence genes and their putative functions related to chemotaxis, adherence, invasion, evasion of the host immune system, and damage of host tissue and cells. Thus, it supports the pathogenicity of *V. echinoideorum*. In conclusion, the interaction of *V. echinoideorum* with injured sea urchin facilitates the development of lesion syndrome and therefore, revealing its potentiality as an opportunistic pathogen.

## Introduction

**Figure 1:**
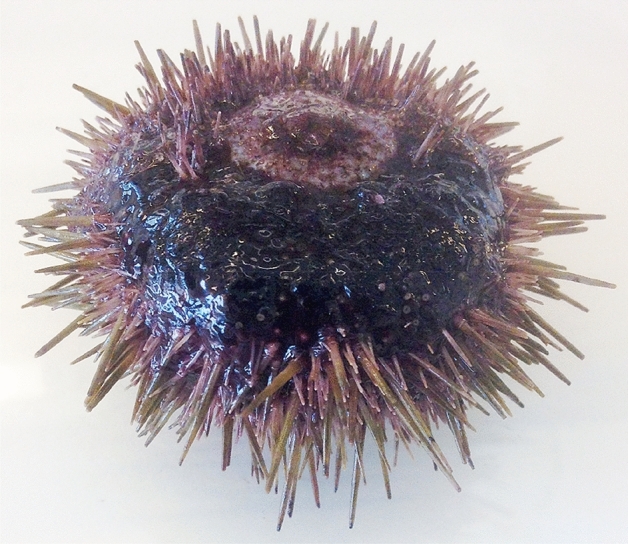
Lesion syndrome of green sea urchin (*S. droebachiensis*) reported in Northern Norway. The animal here is presented up-side down with mouth located on the top.

Globally, there is an increased commercial demand for edible sea urchin principally because of the roe, which is considered as a culinary delicacy^1^. Additionally, sea urchins received much attention as a potential source of antimicrobial^2^, antioxidant, and anti-inflammatory bioactive molecules^3–5^. Due to their high commercial value, the interest in sea urchin aquaculture has augmented around the world^6^. However, sea urchin disease has become a progressing threat to the sea urchin production^7^. Over the last few decades, disease events have been documented in many sea urchin species, including those having commercial importance^7^. The most frequently reported diseases of sea urchins are bald sea urchin disease and other kinds, such as togenukesho, spotting disease, black mouth disease, red spot disease, and lesion syndrome^7,8^. The recurring symptoms of all these diseases are necrotic epidermal tissues covered with a dark-colored mucoid layer, absence of spines, pedicellariae, tube feet, and often the upper layer of the skeleton is partially destructed^7,8^. These diseases are not specific for a particular sea urchin species and might not be caused by a single microbial agent^7,9^. Early studies reported that bald sea urchin disease could be infectious and that the causative agents presumably could be opportunistic bacteria^10–13^. However, parasites such as the amoebae *Paramoeba invadens* have also been found to cause severe damage to the sea urchins^14,15^. Researchers proposed that physical damage^8,11,12^ and the grazing action by the gastropod *Vexilla vexillumi* are vital for the development of the characteristic lesion syndrome in sea urchins^16^. It is well established that *Vibrio* species can act as opportunistic pathogens and often are associated with disease progression in both vertebrates and invertebrates^17–21^. Consequently, a direct or indirect association of *Vibrio* species with bald sea urchin disease would not be surprising. Until now, the *Vibrio* species that have been identified as opportunistic pathogens for diseased sea urchins, are *Vibrio anguillarum*^11^*, Vibrio shilonii, Vibrio splendidus, Vibrio harveyi, Vibrio fortis*^22^, *Vibrio alginolyticus*^13^, *Vibrio coralliilyticus*^23^*.* It is unclear whether these *Vibrio* species act as primary causative agents or secondary opportunistic colonizers^7^ and the virulence features of the opportunistic Vibrios remain elusive. Previous studies have also demonstrated that several unidentified *Vibrio* species are associated with sea urchin disease^7,10^. Recently, a novel *Vibrio* bacteria, *Vibrio echinoideorum* (strain NFH.MB010^T^), was isolated and identified from the hard body shell (test) lesions of the green sea urchin (*Strongylocentrotus droebachiensis*) in Northern Norway (Fig. 1) and documented as a novel member of the Splendidus clade^24^. The lesion syndrome observed in *S. droebachiensis* in Norway, demonstrates similar symptoms as bald sea urchin disease, but differs mainly from other diseases, for example, red spot disease show presence of purple-red viscous spots in the denuded area^7,23^. The lesion syndrome was observed under low temperature condition, hence, the involvement of pathogens speculated could be somewhat different than other sea urchin diseases. Importantly, pathogenic behavior of *V. echinoideorum* towards green sea urchins at low temperature has not been reported elsewhere. Although, several members of Splendidus clade (such as *V. celticus, V. crassostreae*, *V. fortis, V. kanaloae, V. lentus, V. pelagius, V.* *splendidus*-related strains and *V. tasmaniensis*) have already demonstrated their pathogenic relationship with their host (mariculture animals)^19,21,25–27^, the role of *V. echinoideorum* in the development of sea urchin lesion formation and the involvement of its virulence related genes in the disease progression, are yet not known.

Therefore, in the present study, we intend to address two central questions. First, *does the novel V. echinoideorum has any role in the lesion formation of green sea urchin?* Second, *does its genome possess any intriguing virulence features?* Furthermore, employing an in vivo and in vitro bacterial pathogenicity experiment and screening the bacterial genome for virulence factors, enabled to understand the pathogenic potential of *V. echinoideorum*.

## Results and discussion

### *V. echinoideorum* stimulates the lesion formation in wounded sea urchin

**Figure 2:**
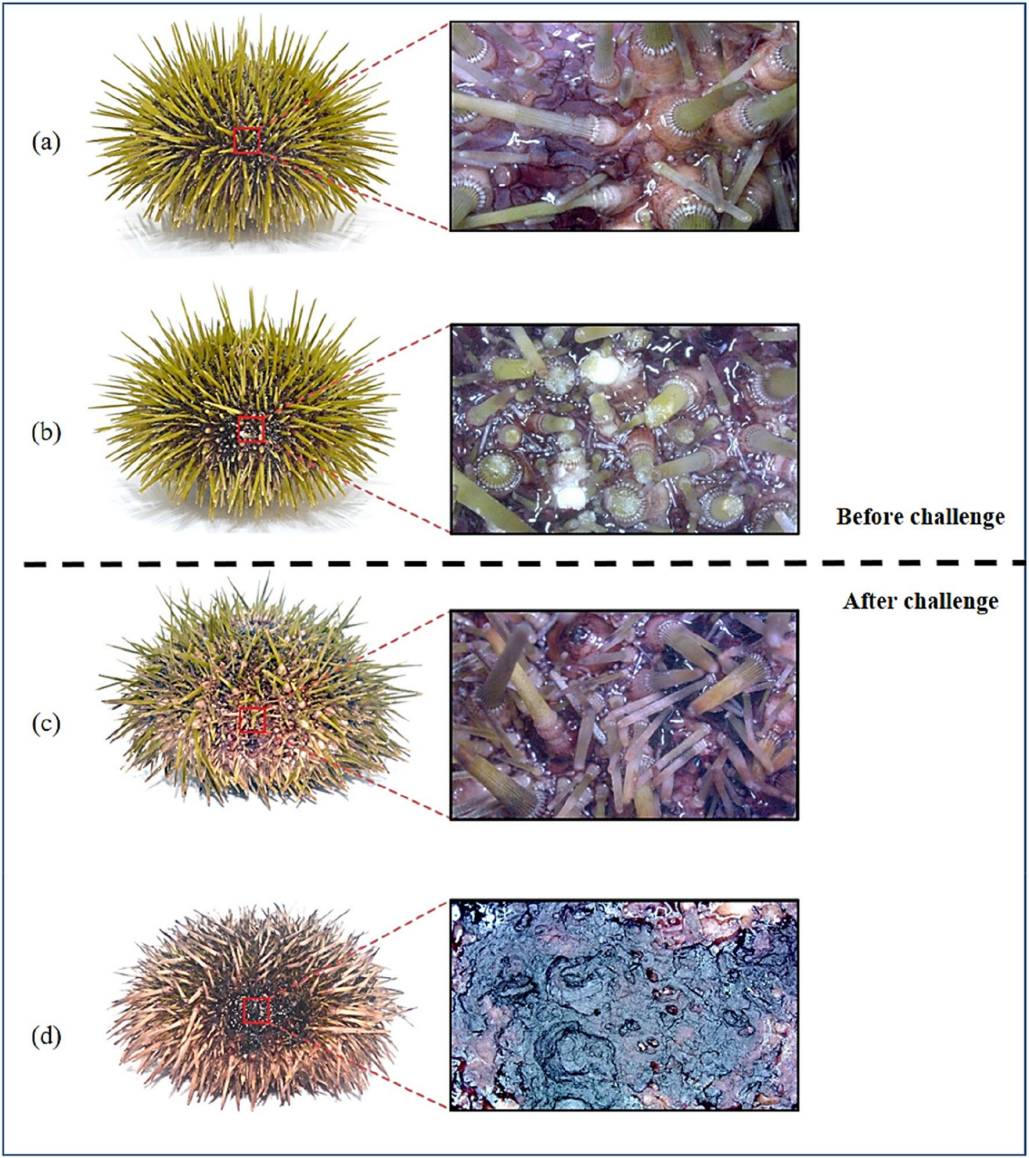
Green sea urchins (*S. droebachiensis*) before (**a**, **b**) and after (**c**, **d**) the in vivo bacterial challenge experiment with *V. echinoideorum* at ± 6–8 °C. (**a**) Healthy sea urchin before the challenge experiment and a closer macroscopic view of intact spines, pedicellariae, tube feet, and other external appendages. (**b**) Artificially wounded sea urchin (spines, pedicellariae, tube feet, and other external appendages were trimmed). (**c**) Recovery of spines, pedicellariae, tube feet, and other external appendages of artificially wounded sea urchin (control, not exposed to bacteria) at the end of the challenge experiment. (**d**) Artificially wounded sea urchin (exposed to bacteria) having necrotized tissues, devoid of spines, pedicellariae, tube feet, and other external appendages.

In vivo bacterial challenge experiment was implemented to address the role of *V. echinoideorum* in the development of sea urchin lesion formation in the presence or absence of artificial wound introduced by mechanical damage. The results from the bacterial challenge experiment are summarized in Table 1. The first symptom of lesion formation appeared in the first week after the bacterial challenge. Generally, a healthy sea urchin possesses greenish healthy spines with intact tissue, outer surface appendages and skeleton covering the whole ambulacral (ab) and interambulacral (Iab) zone on the equator region of the sea urchin test area (Fig. 2a). Only the sea urchins that were mechanically abraded (Mab) around the ambulacral (ab) and interambulacral (Iab) zone (Fig. 2b), developed lesions on the exterior of sea urchin test upon exposure to *V. echinoideorum*. The unexposed Mab animals had no signs of lesions and as time went, spines were regenerated in the artificially wounded areas (Fig. 2c). The non-mechanically abraded (nMab) group exposed to bacteria, also had no characteristic lesions, but rather had crippled tube feet and reddish spines, demonstrating their progression of illness. Besides, the lesions developed in the infected sea urchins resembles the characteristic features of the naturally developed lesions in diseased sea urchins. The infected animals had an extended lesioned area covered with a dark reddish to black mucoid layer surrounded by fragmented material of necrotized tissues and lacking spines, pedicellariae, and tube feet (Fig. 2d). Apart from the lesioned area, the animals had reddish spines and crippled tube feets. Based on the light microscopic study, the lesion materials were ubiquitously loaded with aggregated coelomocytes, mostly of the red spherule cells (RSCs) type (Supplementary Fig. 1).

**Table 1:** Summary of the outcome of an in vivo bacterial challenge experiment with green sea urchins (*S. droebachiensis*) and *V. echinoideorum*.

Feature	Control		Treated	
Group	Mab^a^	nMab^a^	Mab	nMAb
No. of animals	8	8	8	8
Infected animals	0	0	8	0
Wound recoveries	8	NA	0	NA

a = *Mab,* mechanical abrasion; *nMab,* none mechanical abrasion; *NA,* not available.

**Figure 3:**
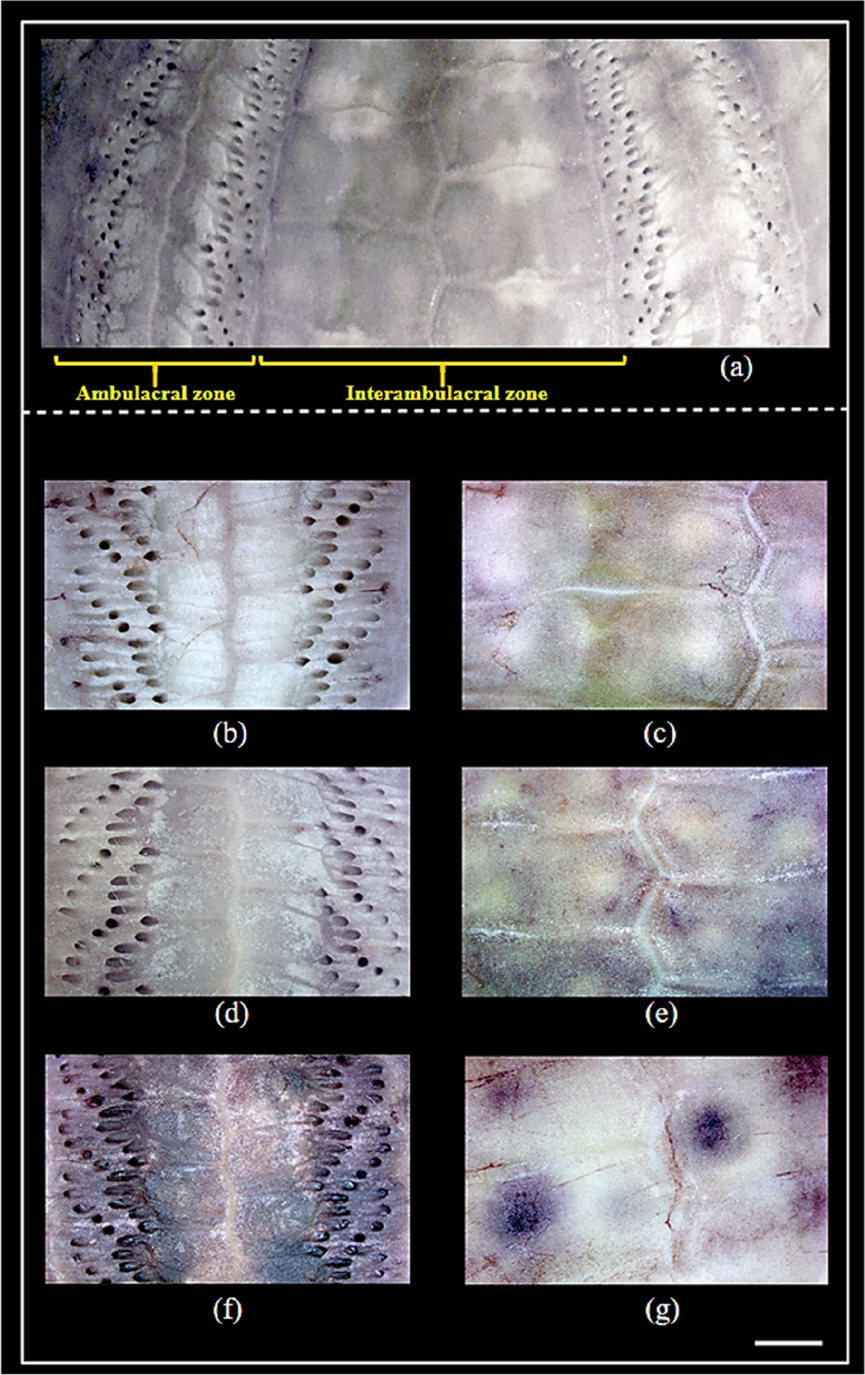
Internal view of green sea urchins (*S. droebachiensis*) hard body shells (test) after the in vivo bacterial challenge experiment with *V. echinoideorum* at ± 6–8 °C. (**a**) Healthy interior of the ambulacral and interambulacral test zone. (**b**) and (**c**) represents the intact ambulacral and interambulacral area of a healthy sea urchins. Here, (**d**) and (**e**) depicts the artificially wounded sea urchins without exposure to bacteria. No signs of calcareous skeleton, blackish patches, swelling of tube feet pores and darkened perforations are seen, whereas (**f**) and (**g**) represents the artificially wounded, bacterial exposed sea urchins with signs of skeletal damage. Scale bar 2 mm.

On the other hand, microscopic studies of the interior of the lesioned sea urchin test revealed the Mab control animals (not exposed to bacteria) had no signs of tissue redness, swelling, and perforations similar to healthy nMab control group (Fig. 3a–e). However, in the infected animals the interior of the lesioned area represents the calcareous skeleton, which was imprinted with blackish patches, and the pores of the tube feet were distinctively swollen and darkened (Fig. 3f).Traces of test decalcification and perforations were clearly visible on the interior side of these lesioned areas (Fig. 3g). In the literature, it is well documented that animals with exposed wounded areas create conditions that often favors the opportunistic *Vibrio* and other bacteria species to become more pathogenic because of the hosts impaired physical defense system^28^. This in vivo bacterial challenge experiment clearly demonstrates that injured animals were highly susceptible to the lesion development in the presence of *V. echinoideorum*. Previous studies on *Strongylocentrotus purpuratus* (purple sea urchin) have shown that experimentally induced lesions in the presence of potential pathogens (*V. anguillarum* and *Aeromonas salmonicida*) were not fatal. This is presumably because the sea urchins after some days, were able to recover from the artificially induced lesions and the regeneration of spines, and exterior test tissues were visible^11^. *S. droebachiensis* also have demonstrated a similar phenomenon of healing from the experimentally induced lesions^12^. Earlier, an artificially induced lesion in green sea urchin has been categorized in three stages- (1) denuded test with light pigmented, thin mucoid layer, (2) dark pigmented, thick mucoid layer, and (3) reduction of lesion size with regrowth of spine and other external appendages on the test^12^. It has been speculated that expansion of the lesioned area may lead to perforation of the test, which is lethal for sea urchins^11,12^. The lesions developed in the presence of *V. echinoideorum* expanded in area as the number of days increased. Additionally, bacterial re-isolation from the undiluted lesion material from 8 out of 8 infected animals resulted in pure cultures of *V. echinoideorum*. Approximately 10^6^–10^7^ CFU/2 µl was recovered from the lesion material of infected sea urchins. The *V. echinoideorum* taxonomic identity was confirmed by the MALDI-TOF MS. In this study, matching score > 2.3 against in-house *Vibrio* mass spectral database were considered as the confirmation of species identity. The generated score for the reisolated strains were > 2.3, and thus verified the identity of the *V. echinoideorum*. Re-isolation and confirmation of *V. echinoideorum* from the challenged infected animals provide a strong indication of the importance of *V. echinoideorum* in the development of lesions. Overall, the lesioned areas (extended from ambulacral to interambulacral zone) of infected sea urchins are accompanied by a high concentration of *V. echinoideorum*, conspicuous mucoid tissue redness and perforation, inability to recover spines, pedicellariae, tube feet, and other appendages. The present study suggests that *V. echinoideorum* can be lethal to the wounded sea urchins and may lead to a mortality event.

### Cytolytic virulence effect on host immune cells

**Figure 4:**
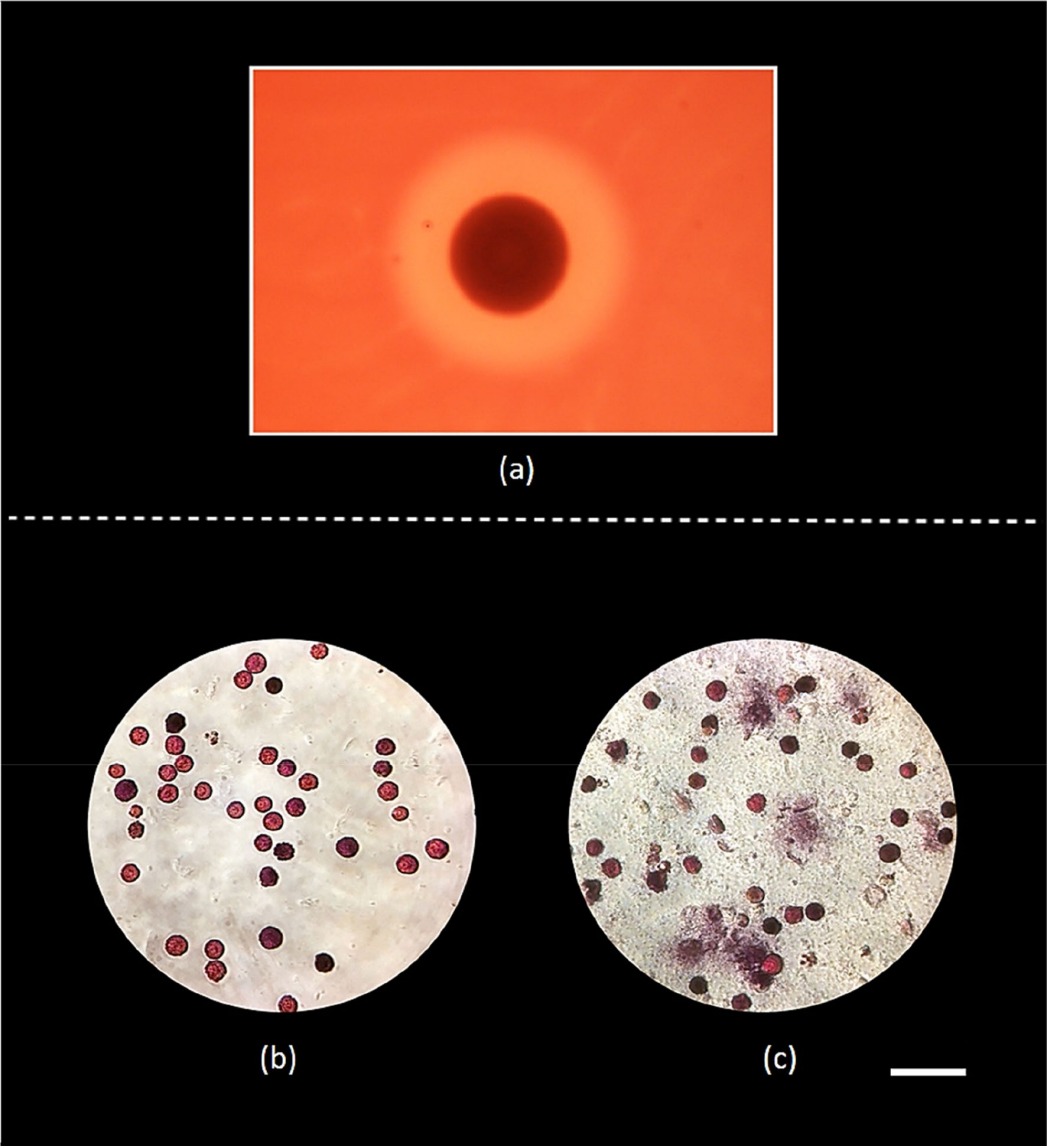
Cytolytic properties of *V. echinoideorum* towards sheep RBCs and sea urchin RSCs. (**a**) Hemolysis reaction against sheep RBCs is presented here with a clear zone around the colony (16 mm in diameter zone) on blood agar. (**b**) Control with RSCs not exposed to bacteria, (**c**) RSCs exposed to bacteria. Scale bar 60 µm.

Cytolysis is an important feature of pathogens invasion strategy during interaction with hosts. Though in vivo challenge experiment demonstrates the detrimental role of *V. echinoideorum,* this does not elucidate its virulence behavior towards sea urchin immune cells. Thus, hemolysis and spherulolysis activity assessment of *V. echinoideorum* was crucial to elucidate its virulence characteristics towards host immune cells. Hemolytic assessment on sheep red blood cells (RBC) was the first step to understand whether *V. echinoideorum* possess cytolytic potentiality or not. In this study, the assay demonstrates a clearance zone of 16 mm in diameter around the bacterial colony of *V. echinoideorum*, confirms the strong β-hemolysis (beta hemolysis) activity on sheep RBCs (Fig. 4a). This was the first indication, which supports that *V. echinoideorum* has the capability of damaging the host cells by its cytolytic toxins. Next, this cytolytic property of *V. echinoideorum* was intended to assess against the sea urchin RSCs, an important immune cell of sea urchin. In this study, RSCs were found abundant in the lesion materials of diseased animals. It has been reported earlier that RSCs are found to have a strong response to foreign invaders^29^. Thus, RSCs are considered to be a good candidate for cytolytic experiment. Prior to in vitro challenge experiments, post sorted RSCs were observed to be ~ 90% viable as confirmed by trypan-blue exclusion method. Next, we observed that the unexposed RSCs (control) were intact (Fig. 4b), whereas RSCs exposed to *V. echinoideorum* were cytolysed and released pigments to the surroundings (Fig. 4c). Therefore, RSCs cytolysis capability of *V. echinoideorum* indicates its cytolytic potential towards sea urchin cells, substantiating its invading properties towards the host cellular defense systems.

### Putative virulence related genes

**Figure 5:**
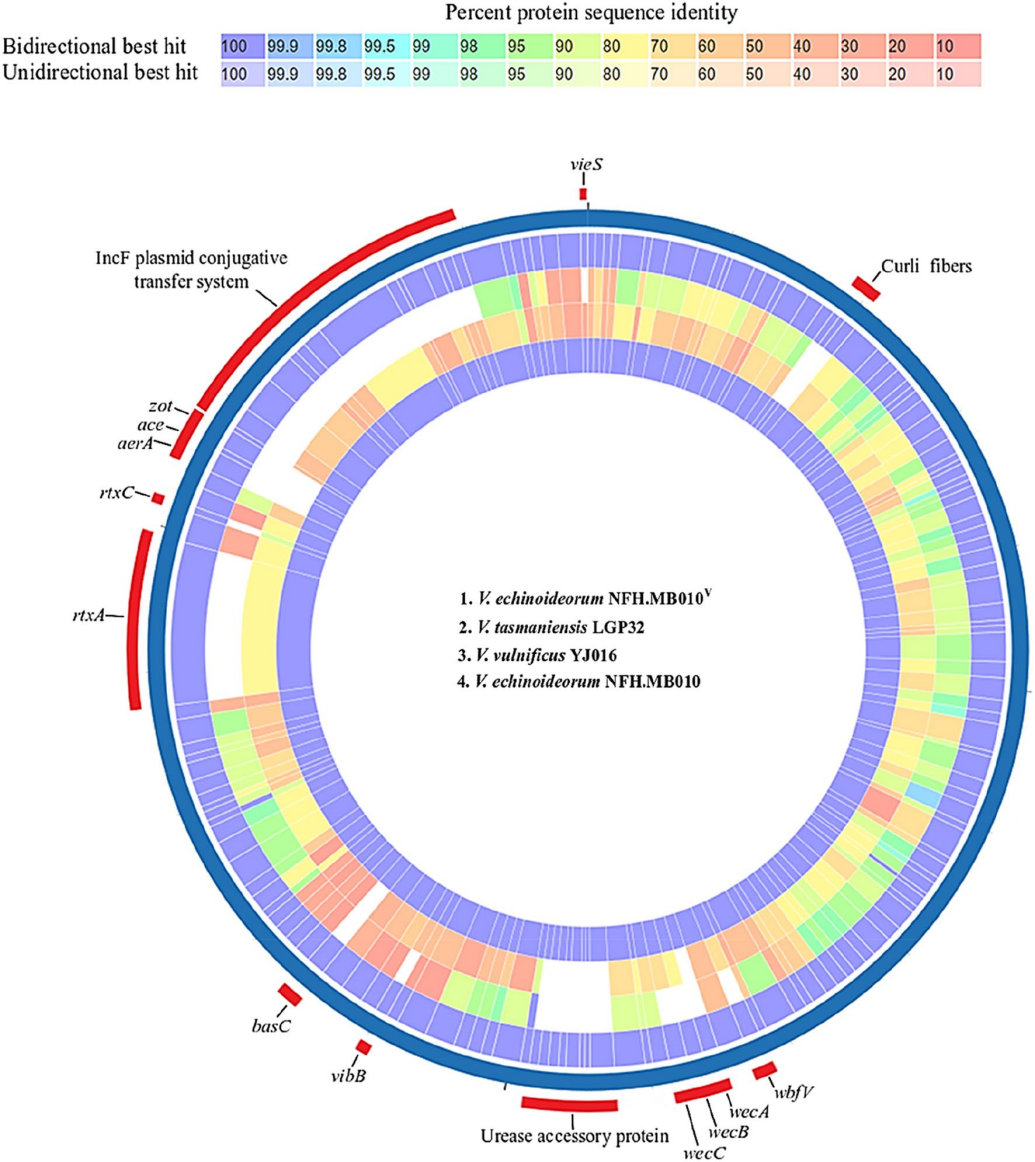
A circos plot featuring the proteome comparison of predicted virulence genes of *V. echinoideorum*. From the outer ring to the inner ring: *V. echinoideorum*, *V. tasmaniensis* LGP32, *V. vulnificus* YJ016, and *V. echinoideorum* (as control replicate). Only distinguished features between these strains are labelled.

Both in vivo and in vitro challenge experiments demonstrates the pathogenic role of *V. echinoideorum*. One of the objectives of this study is to explore the virulence features that *V. echinoideorum* possess. For this purpose, genome mining and proteome comparison was implemented to locate the presence and absence of gene products known to have virulence roles during vibriosis. Two well-known marine *Vibrio* pathogens, *V. tasmaniensis* LGP32 and *V. vulnificus* YJ016, were included in this comparative study. The outcome of the genomic mining of *V. echinoideorum* by the RAST and VFanalyzer comprises predicted virulence genes, summarized in Table 2. The circos plot generated by the PATRIC proteome comparison tool depicts the distinguishing virulence features between the proteomes of *V. echinoideorum*, *V. tasmaniensis* LGP32, and *V. vulnificus* YJ016 (Fig. 5). The percent identity of amino acid (AA) sequences between the subjected species generated by the PATRIC proteome comparison tool is provided in the Supplementary datafile 1. Based on this comparison, accessory cholera enterotoxin (*ace*) and zonula occludens toxin (*zot*) genes were uniquely detected in the *V. echinoideorum* genome. To understand if these *ace* and *zot* genes are affiliated with any prophage system, the analysis performed by the PHASTER tool identified 2 questionable prophage regions of length 28.7 kb and 13.9 kb (Supplementary Fig. 2). The 28.7 kb prophage region 1 was related to a Mu-like phage and featured 39 protein-coding sequences (CDSs). The 26 CDSs in the 13.9 kb prophage region 2 on the other hand, were predicted to be related to different *V. cholerae* phages, including filamentous phages like KSF-1ϕ, VEJϕ, and VCYϕ. Additionally, a bacteriophage f237 related sequence was predicted in this prophage region 2. The *zot* gene is located in this prophage region 2. Its AA sequence was found to be 52% identical to *V. cholerae* phage KSF-1ϕ based on a BLASTP search. Both prophages were comprised of many hypothetical proteins with unassigned function. The presence of these prophages suggests that the affiliated gene function may contribute to the adaptations of *V. echinoideorum* in the host. Additionally, the VieSAB three-component system related gene sequences of the *V. echinoideorum* genome, was found to have more AA sequence identity with the *V. vulnificus* YJ016 gene products than with the ones of *V. tasmaniensis* LGP32. Interestingly, the multifunctional-autoprocessing repeats-in-toxin (*rtxA*) and cytolysin-activating lysine-acyltransferase (*rtxC*) present in the *V. echinoideorum* genome had AA sequence identities of 85% and 90%, respectively, to the RtxA and RtxC protein of *V. vulnificus* YJ016. The *rtxA* and *rtxC* genes were not detected in the *V. tasmaniensis* LGP32 genome. Surprisingly, acid resistance urease related genes were observed in the genome of *V. echinoideorum*, which were absent in the other two reference species. The estimated AA sequence identity of the IncF plasmid conjugative transfer related proteins were 50–60% when compared to *V. vulnificus* YJ016. The homologous genes related to IncF plasmid conjugative transfer were not found in the *V. tasmaniensis* LGP32 genome. Among these compared species, only *V. echinoideorum* proteome had the gene product related to the curli fiber biogenesis. Additionally, differences in the anti-phagocytosis and iron uptake-related virulence genes between the species were also observed. The vibriolysin protein of *V. echinoideorum* had 95% AA sequence identity to the vibriolysin of *V. tasmaniensis* LGP32 and 70% identity to *V. vulnificus* YJ016. The outer membrane porin (OmpU) of *V. tasmaniensis* LGP32 was 47% similar in AA sequence to OmpU of *V. echinoideorum*. Presence of a transcriptional activator like gene (*toxR*) was observed in the *V. echinoideorum* genome, its product had 29% and 32% AA sequence identity to their homologous of *V. tasmaniensis* LGP32 and *V. vulnificus* YJ016. This *toxR* gene was detected adjacent to an aerolysin-like enterotoxin (*aerA*) related gene in the *V. echinoideorum* genome while *aerA* like genes were not detected in the genome of *V. tasmaniensis* LGP32 and *V. vulnificus* YJ016. The thermolabile hemolysin (Tlh) of *V. echinoideorum* was found to have 93% and 70% AA sequence identity to the similar protein of *V. tasmaniensis* LGP32 and *V. vulnificus* YJ016. Several virulence regulatory genes were found in the *V. echinoideorum* genome in addition to the VieSAB system. Additionally, the BarA-UvrY two-component virulence regulatory system was observed having 99% and 93% AA sequence identity to its homologous gene product identified in the *V. tasmaniensis* LGP32 and *V. vulnificus* YJ016 genome. Overall, the virulence genes predicted in the *V. echinoideorum* genome were found related to the chemotactic motility, adhesion, anti-phagocytosis, acid resistance, oxidative stress response, iron sequestering system, quorum sensing, toxins and its secretion system, conjugative transfer system, and genes regulating virulence functions. The above-mentioned virulence features of *V. echinoideorum* explicitly suggest that this bacterial strain is well equipped with the virulence factors that may enable the bacteria to have a strong interaction with sea urchins and cause severe health problems.

**Table 2:** Overview of putative virulence genes identified in the *V. echinoideorum* genome.

Virulence gene	Annotation
**Chemotaxis and motility**	
*cheA, cheB, cheR, cheV, cheW, cheY, cheZ,*	Chemotaxis
*flgA, flgB, flgC, flgD, flgE, flgF, flgG, flgH, flgI, flgJ, flgK, flgL, flgM, flgN,* *flaA, flaC, flaD, flaF, flaG, flaI* *flhA, flhB, flhF, flhG* *fliA, fliD, fliE, fliF, fliG, fliH, fliI, fliJ, fliK, fliL, fliM, fliN, fliP, fliQ, fliR, fliS,* *flrA, flrB, flrC,* *motA, motB, motX, motY,*	Flagella
**Adherence**	
*mshA, mshB, mshC, mshD, mshE, mshF, mshG, mshH, mshI, mshJ, mshK, mshL, mshM, mshN*	Mannose-sensitive hemagglutinin (MSHA type IV pilus)
*pilA, pilB, pilC, pilD,*	Type IV pilus
*csgE, csgF, csgG*	Curli fibers
**Anti-phagocytosis**	
*rmlA, rmlB, rmlC, rmlD, wbfB, wbfU, wbfY, wbfV/wcvB, wza, wzb, wzc, wecA, wbjd/wecB, wecC*	Capsular polysaccharide (CPS) component
**Acid resistance**	
*Ure αβγ*	Urease subunits
*ureD, ureE, ureF, ureG, ureJ*	Urease accessory protein
**Oxidative stress response**	
*sodB*	Protection from Reactive Oxygen Species
**Iron uptake**	
*vibA, vibB, vibC, vibE*	Vibriobactin related
*vctA*	Enterobactin receptors
*hutA, hutR*	Heme receptors
*vctC, vctD, vctG,*	Periplasmic binding protein-dependent ABC transport systems
*barB, basC, basG, bauB, bauC, bauD*	Acinetobactin related
**Quorum sensing**	
*luxS, cqsA*	Autoinducer 2 (AI-2)
**Secretion system**	
*epsC, epsD/gspD, epsE, epsF, epsG, epsH, epsI, epsJ, epsK, epsL, epsM, epsN*	EPS type II secretion system
**Toxin**	
*vvp/vsm*	Vibriolysin, extracellular zinc metalloendopeptidases
*ompU*	Outer membrane protein (porin)
*rtxA, rtxB, rtxC, rtxD*	RTX toxin
*tlh*	Thermolabile hemolysin
*aerA/act*	Aerolysin AerA/Cytotoxic enterotoxin Act
*ace*	Accessory cholera enterotoxin
*zot*	Zona occludens toxin
**Conjugative transfer**	
*traA, traB, traC, traD, traE, traF, traH, traI, traK, traL, traN, traU, traV traW,*	IncF plasmid conjugative transfer protein
*trbC*	
**Virulence regulation**	
*gacA, gacS*	GacS/GacA sensor/kinase two-component regulatory system
*vieS, vieA, vieB*	VieSAB signal transduction system
*toxS*	Transmembrane regulatory protein for enterotoxin
*toxR*	Transcriptional activator for enterotoxin
*rstA*	Two-component regulatory system

## Conclusion

In conclusion, the evidence provided by this bacterial challenge study suggests that *V. echinoideorum* demonstrates a detrimental impact on the injured green sea urchins leading to the symptoms seen in the lesion syndrome. Additionally, the virulence determinants identified in *V. echinoideorum* genome support the virulence capability that define the bacteria as an opportunistic pathogen. The present study does not infer that *V. echinoideorum* is the causative agent of lesion syndrome observed in the green sea urchins in Northern Norway, but rather imply that it may play a crucial role in a polymicrobial infection in cooperation with other opportunistic pathogens. To the best of our knowledge, limited information is available regarding the virulence features of *Vibrio* species associated with sea urchin disease. Therefore, further study in elucidating the function of the above-mentioned virulence genes associated with the development of green sea urchin lesion syndrome, could be crucial for understanding the vibriosis in echinoderms and other commercially important marine animals. Finally, the infectomics study on *V. echinoideorum*-sea urchin interaction at low temperature may serve as a good in vivo model for a comprehensive study on temperature dependent pathogenicity of opportunistic *Vibrio* pathogens.

## Materials and methods

### Ethical statement

Green sea urchins used in this study do not belong to a group of endangered species and are not subjected to any ethical restrictions regarding their use for research purposes. For the experimental procedures, sea urchins were handled according to the guidelines provided by the UiT The Arctic University of Norway and the Aquaculture Research Station in Tromsø, Norway.

### Sea urchin

Healthy green sea urchins were collected along the northern coast of Norway and maintained in water tanks facilitated with recirculating seawater system, before they were transported to the laboratory (Aquaculture Research Station, Tromsø) for the bacterial challenge experiment. Healthy individuals of approximately 4–5 cm in diameter and 45–55 g in body weight were used in this experiment. The animals were kept in aerated seawater at ± 6–8 °C under light/dark conditions.

### Bacterial strains and growth conditions

*V. echinoideorum* was cultured on modified marine agar (FMAP: 15 g L^−1^ Difco Marine broth, 5 g L^−1^ peptone supplemented with 30% sterile seawater) at 12 °C for 72 h. For the experimental purpose, bacteria were cultured to the exponential growth phase in FMAP broth overnight at 12 °C under a moderate shaking.

### In vivo bacterial challenge experiment

The pathogenicity of *V. echinoideorum* was assessed by a two-step immersion method. Prior to the experimental challenge, healthy green sea urchins were acclimatized in a water tank facilitated with a system of recirculated, UV treated, and filtered seawater adjusted to a low water flow rate and oxygen saturation > 100%. The tank temperature was controlled and kept at ± 6–8 °C to mimic the local seawater temperature. The sea urchins were acclimated for 10 days under light/dark conditions. They were fed artificial sea urchin feed (kindly provided by Nofima, Tromsø, Norway) once or twice a week and examined daily for any symptoms of deteriorating health signs before the challenge experiment occurred. The sea urchins were divided into two groups before the experiments: Mechanically abraded (Mab) and non-mechanically abraded (nMab). Both groups were further subdivided into two subgroups: Control (unexposed) and treated (exposed to bacteria). Eight animals were allocated to each group. A sterile fine-point iris scissor was used for mechanically trimming and scraping off the spines, tube feet, and other appendages covering approximately 1 × 1 cm of the ambulacral and interambulacral zone on the equator region of the sea urchin test area. On the bacterial challenge day, sea urchins from both the Mab and nMab groups were transferred from the observation tank to a plastic container and then submerged into a bacterial suspension (concentration of bacteria was adjusted to approximately ~ 10^8^ CFU/ml). Next, these plastic containers with sea urchins in a bacterial suspension, were placed in a temperature-controlled refrigerator (maintained at ± 6–8 °C) supplemented with oxygen saturation near to 100%. Sea urchins in all infection groups were allowed to remain in the bacterial suspension for 3–4 h, and after the treatment, they were returned to the observation tank with gentle handling. All sea urchins were kept in the aerated filtered seawater for up to ~ 30 days. They were examined daily for any lesion development and death of individuals. The bacterial concentrations were determined in the lesion materials (equivalent to 2 µl) from infected sea urchins. Later, the developed lesion areas were subjected to microscopic examination using Zeiss SteREO Discovery.v20 to observe any signs of skeletal damage leading to decalcification and perforation on the test interior of the infected sea urchins. Prior to the microscopic examination of the test interior, the lesioned areas were excised, and tissue debris present in the test interior was carefully washed with sterile seawater. All images captured by the microscope were processed with Cytosketch (Cytocode) and Photoshop CS6 (Adobe) imaging software.

### Bacteria re-isolation and MALDI-TOF MS fingerprinting

Bacteria were re-isolated from undiluted lesion materials by plating on FMAP and incubation at 12 °C for 72 h. Next, the identity of *V. echinoideorum* was confirmed by matrix-assisted laser-desorption/ionization time of flight mass spectrometry (MALDI-TOF MS) analysis. Prior to the verification process, an in-house bacterial reference library was built comprising mass spectra profiles obtained from several *Vibrio* species that were previously isolated from healthy and diseased green sea urchins. *V. echinoideorum* MALDI-TOF spectra were also added to the bacterial reference mass spectra library. To construct the reference library, 5 colonies (biological replicates) from a pure culture were transferred to FMAP broth and were grown overnight. Next, 1 ml of the overnight grown culture from the individual colonies were centrifuged at 10,000 g for 2 min. After centrifugation, pellets were washed with 1 ml ultra-pure water and then centrifuged at 10,000 g for 2 min. Supernatants were discarded and the pellets were thoroughly suspended in 300 µl of ultra-pure water followed by 900 µl 96% ethanol. The samples in the water–ethanol solution were vortexed and centrifuged at 10,000 g for 2 min. Next, the bacterial lysates were prepared by adding 50 µl of 70% formic acid to the bacterial pellets followed by 50 µl of undiluted acetonitrile. Samples were mixed thoroughly again by vortexing and centrifuged at 10,000 g for 2 min. Supernatants (protein extracts) were transferred to fresh tubes for further MS analysis. For each biological replicate, 5 technical replicates of extracted protein samples were spotted, which resulted in a total of 25 spots for the analysis. From these 25 spots, 25 independent mass spectra were generated for individual bacterial species. A volume of 1 µl of each sample was pipetted onto a clean, 96 target main-spectrum (MSP) polished steel plate. The samples were air-dried at room temperature and subsequently overlaid with 1 µl of α-cyano-4-hydroxy-cinnamic acid (HCCA) matrix solution. Next, Microflex LT MALDI-TOF mass spectrometer (Bruker Daltonics, Germany) was used to record raw mass spectra of molecules from individual spots. Bacterial test standard (Bruker Daltonics, Germany) was used for the calibration purpose. Spectra acquisition was performed using Flex Control 3.4 software (Bruker Daltonics, Germany) with default parameter settings (positive linear mode with ion source 1: 19.98 kV; ion source 2: 18.08 kV; lens voltage, 6 kV; delay time of 120 ns and laser frequency of 60.0 Hz; mass range, 2000–20,000 Da). Technically, 240 laser shots in 40-shot steps from different areas of the spotted samples were accumulated and analyzed for each mass spectrum under the automatic mode, according to the manufacturer’s procedure (Bruker Daltonics, Germany). Independently generated spectrum was further processed (smoothing method, Savitski-Golay; baseline subtraction method, multipolygon; normalization method, maximum normalization) with Bruker MALDI Biotyper Compass Explorer 4.1 software (Bruker Daltonics, Germany) to create the MSP in-house reference library and to capture the true biological variability of a bacterial organism. The Biotyper software utilizes a pattern-matching approach that performs true statistical multivariate analyses on the data, including peak positions, intensities, and frequencies. Subsequently, the analysis compares the peak list between the sample organisms and the organisms of the reference library, resulting in logarithmic score values between 0.00 and 3.00. This value reflects the similarity between the mass spectrum of reference and the sample organisms; the higher the score, the more likely there is a match between the sample organisms and the organisms in the reference library. According to the manufacturer (Bruker Daltonics, Germany), the specified value for a reliable identification at the species level is set to a score of ≥ 2.3. A score from 2.299 to ≥ 2.0 represents a high probability for the same species, from 1.999 to ≥ 1.7 a high probability for the same genus, and a score < 1.7 is considered unreliable as species taxonomic identification.

### Cytolytic virulence assessment

Cytolytic virulence activity of *V. echinoideorum* was determined by an agar-based hemolysis assay (breakdown of sheep red blood cells) and spherulolysis reactions (cytolysis of red spherule cells). Hemolysis was determined by pipetting 5 µl of overnight grown bacterial culture onto the center of a FMAP blood agar plate (FMAP supplemented with 5% defibrinated sheep blood) and incubated at 12 °C for 72 h. The spherulolytic reaction was performed on the pure population of RSCs isolated from healthy sea urchins. By using the Fluorescence-activated cell sorting (FACS) technology, the RSCs were isolated and sorted into a 48 well plate (10^5^ cells/well)^30^. Filtered cell-free coelomic fluid (FCF_CF_) supplemented with 10 mM CaCl_2_ and 10 mM HEPES was pre-added to each well prior to RSCs sorting and later, the total volume was adjusted to 300 µl (if needed, FCF_CF_ was used). The viability of the sorted cells was determined by trypan-blue exclusion (0.2% v/v, final concentration) using FastRead counting slides (Immune Systems Ltd). Immediately after sorting, the purity of the RSCs population was validated microscopically using Leica DM6000. Prior to the bacterial challenge, the wells were divided into two groups: Control (unexposed) and treated (exposed to *V. echinoideorum*). The treated samples were challenged with *V. echinoideorum* at a concentration adjusted to approximately a multiplicity of infection (MOI) of 100 bacteria per RSCs. Next, the challenged RSCs were incubated at 6 °C for 24 h. Control and treated wells were inspected for RSCs cytolysis using a Zeiss Axiovert 40 CFL microscope. Color images were captured with an iPhone 6s connected to the ocular lens by an eyepiece adapter and attached to an additional macro zoom lens (14x, olloclip). The captured images were processed with Cytosketch (Cytocode), Fiji, and Photoshop CS6 (Adobe).

### Genomic mining for putative virulence factors

The assembled genome of *V. echinoideorum* (Assembly: GCA_004764665.1) was subjected to virulence gene prediction and annotation by using both the RAST annotation server^31,32^ and virulence factor database (VFDB)^33^. Resulted putative virulence factors were compared in silico using the online bacterial virulence factor identification tool named VFanalyzer, available in the VFDB^33^. *V. vulnificus* YJ016 (Assembly: GCA_000009745.1) was selected for comparative analysis in VFDB. Prominent virulence genes of *V. echinoideorum*, *V. tasmaniensis* LGP32 (Assembly: GCA_000091465.1), and *V. vulnificus* YJ016 bacterial species were compared using a proteome comparison tool provided by The Pathosystems Resource Integration Center (PATRIC) (https://www.patricbrc.org)^34^. The comparative proteome analysis was performed using the parameter, minimum sequence coverage- 10%, minimum identity- 10% and E value- 1e^−5^. Furthermore, potential prophage-like regions present in the *V. echinoideorum* genome were predicted using the prophage identification and annotation tool called PHASTER (Phage Search Tool Enhanced Release)^35,36^.

## Supplementary Information

Supplementary Information 1.

Supplementary Figures.
