# Supplementary material for: Evidence for association of Vibrio echinoideorum with tissue necrosis on test of the green sea urchin Strongylocentrotus droebachiensis
Source: Sci Rep. 2022 Mar 22;12:4859. doi: 10.1038/s41598-022-08772-2 (PMC8940906; doi:10.1038/s41598-022-08772-2)
Supplement: Supplementary file 2 — Supplementary Figures. [file 41598_2022_8772_MOESM2_ESM.pdf]

Evidence for association of *Vibrio echinoideorum* with tissue necrosis on test of the green sea urchin *Strongylocentrotus droebachiensis*

Jonathan Hira[1] and Klara Stensvåg[1*]

[1]The Norwegian College of Fishery Science, The Faculty of Biosciences, Fisheries and Economics, UiT The Arctic University of Norway, Tromsø, Norway.

***** Correspondence: klara.stensvag@uit.no

Supplementary figures

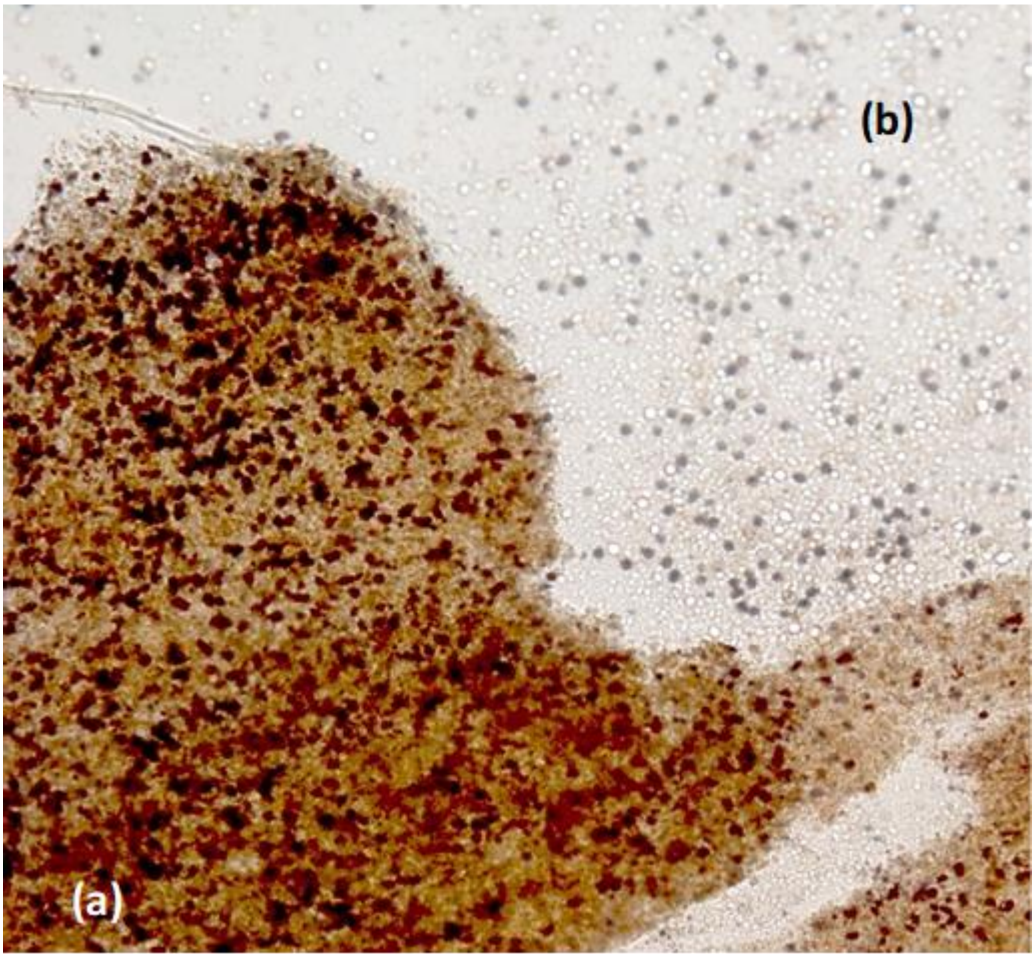

Supplementary figure 1: Light microscopic image of diseased green sea urchins (*S. droebachiensis*) lesion materials. (a) lower left of the image represents fragmented lesion materials visualized under a light microscope (total magnification 50X). Lesion material consists of aggregated red and other cell types of sea urchin. Red spherule cells are the most abundant cell type seen here and observed to be cytolysed. Release of their pigment materials results in generating the dark reddish color of lesion material. (b) upper right corner of the image represents the disintegrated cells from the aggregated materials.

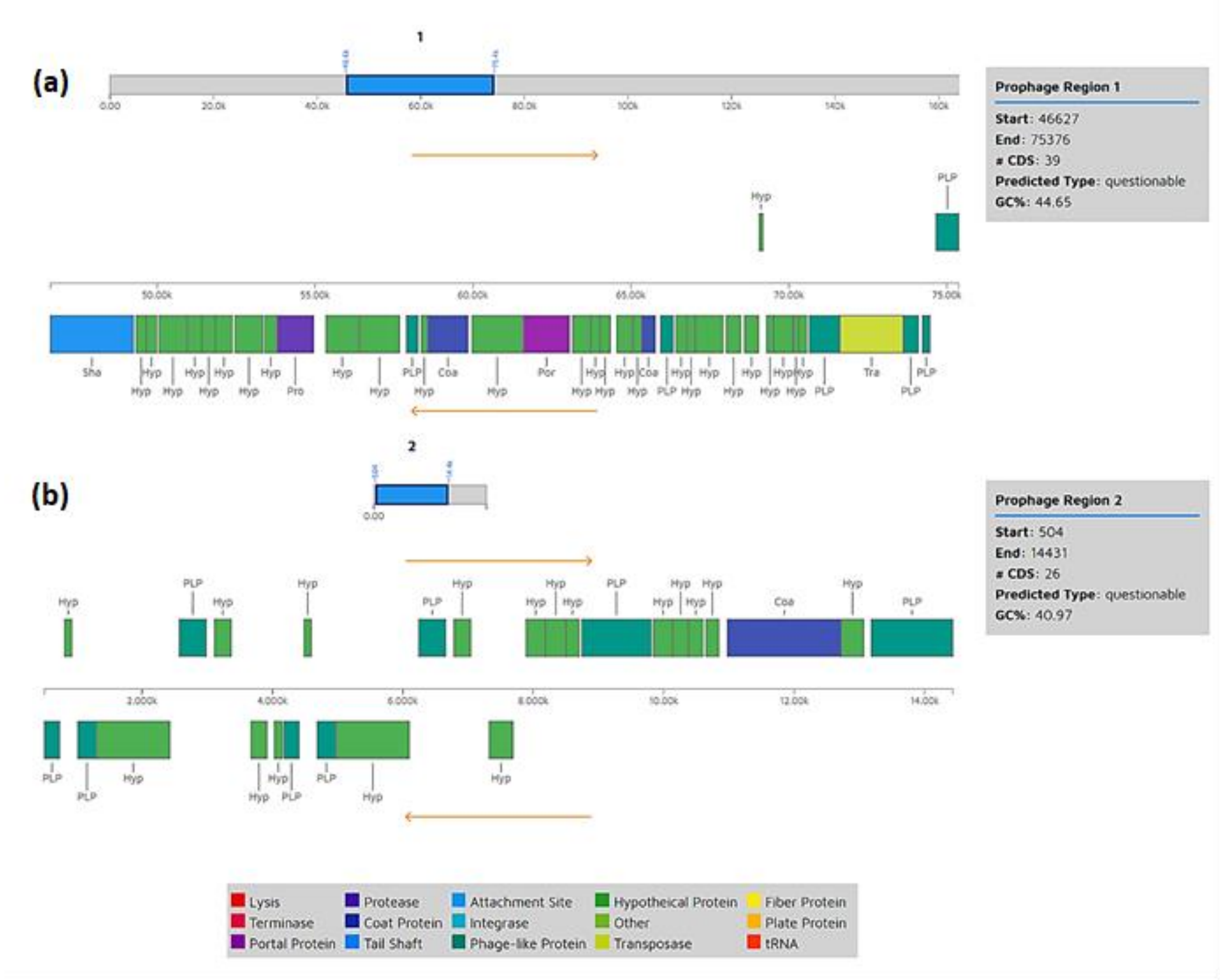

Supplementary figure 2: (a) Prophage region 1 (28.7 Kb) and **(b)** region 2 (13.9 Kb) observed in the genome of *V. echinoideorum*, predicted by PHASTER tool. Prophage regions 1 and 2 contain 39 and 26 coding sequences. Coding sequences are illustrated as colored boxes according to their functional role. Prophage 1 is found related to Mu-like phage and prophage 2 has functional relevance to different *V. cholerae* phages, including filamentous phages like KSF-1ϕ, VEJϕ, and VCYϕ. Additionally, bacteriophage f237 related sequences are present in prophage 2 region.
